# Exploring the roles of microbes in facilitating plant adaptation to climate change

**DOI:** 10.1042/BCJ20210793

**Published:** 2022-02-04

**Authors:** Elle M. Barnes, Susannah G. Tringe

**Affiliations:** 1 Department of Energy Joint Genome Institute, Lawrence Berkeley National Laboratory, Berkeley, CA, U.S.A.; 2 Environmental Genomics and Systems Biology Division, Lawrence Berkeley National Laboratory, Berkeley, CA, U.S.A.

**Keywords:** climate change, fabricated ecosystems, microbiome, plant biology, plant–microbe interactions, synthetic communities

## Abstract

Plants benefit from their close association with soil microbes which assist in their response to abiotic and biotic stressors. Yet much of what we know about plant stress responses is based on studies where the microbial partners were uncontrolled and unknown. Under climate change, the soil microbial community will also be sensitive to and respond to abiotic and biotic stressors. Thus, facilitating plant adaptation to climate change will require a systems-based approach that accounts for the multi-dimensional nature of plant–microbe–environment interactions. In this perspective, we highlight some of the key factors influencing plant–microbe interactions under stress as well as new tools to facilitate the controlled study of their molecular complexity, such as fabricated ecosystems and synthetic communities. When paired with genomic and biochemical methods, these tools provide researchers with more precision, reproducibility, and manipulability for exploring plant–microbe–environment interactions under a changing climate.

## Introduction

**Figure 1.**
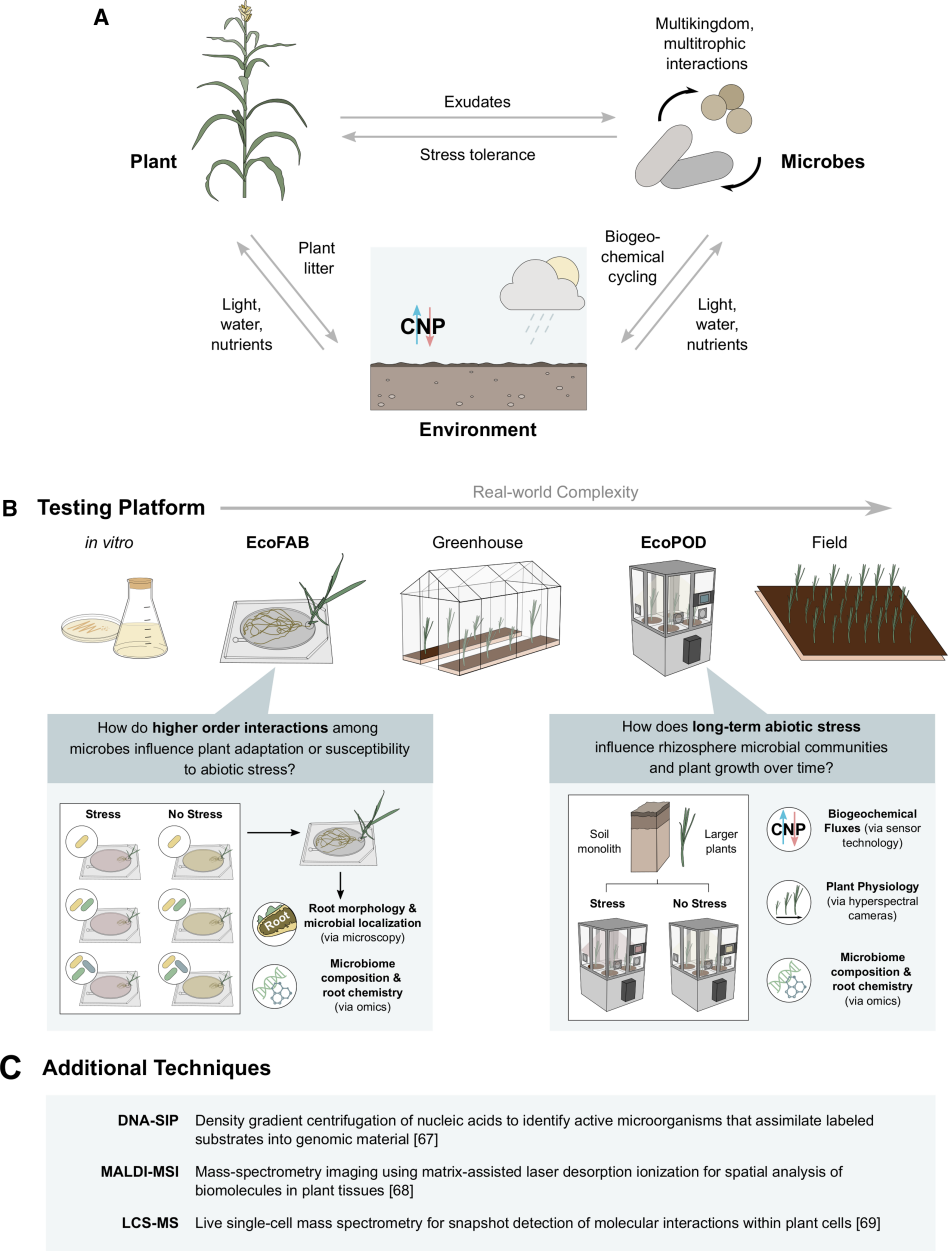
Untangling plant–microbe interactions under climate change (**A**) Network displaying the interactions among plants, microbes, and the environment that can be influenced by climate change. (**B**) Testing platforms currently used to uncover the specific mechanisms controlling these interactions are displayed in increasing real-world complexity. We highlight two new fabricated ecosystem technologies (EcoFABs and EcoPODs) that in conjunction with SynComs bridge existing platforms. Below each new tool, we provide an example of the type of question that can be asked using these technologies as well as the potential data generated. (**C**) Additional experimental techniques that can be used alongside fabricated ecosystems and SynComs.

Climate change has altered ecosystems around the globe by shifting environmental conditions. In addition to global elevated CO_2_ levels, many ecosystems will continue to experience rising temperatures as well as changes to light, humidity, and nutrient and water availability [1]. Drought and flooding events are anticipated to increase, drastically altering the terrestrial ecosystems that we rely on for food and other resources [2]. Plants, as sessile organisms, cannot escape these environmental changes and evidence suggests their adaptation is aided by their close association with soil microbes [3–6]. However, much of what we know about plant stress responses is based on studies where the microbial partners were uncontrolled and unknown [7]. Soil serves as the regional pool that supplies microorganisms to plant roots, and it will respond to climate change both directly via changes in temperature and indirectly through changes in soil edaphic factors such as pH and nutrient concentrations [8,9]. Thus, climate change will likely alter the composition of and interactions between plant and soil communities through a variety of mechanisms that may be plant-mediated, microbe-mediated, or governed by environmental factors (Figure 1A). Teasing apart the contributions of these factors in plant adaptation to climate change could open new doors to improved plant and ecosystem resilience [10]. In this perspective, we highlight some of the key factors influencing plant–microbe interactions under stress and suggest new tools for untangling their complexity.

## Plant-mediated mechanisms

Climate change is expected to substantially impact the composition and structure of plant communities across the globe [1]. Little is known about how these changes will feed back into plant–microbe interactions as plants establish themselves in new locations with potentially different soil microbiomes. In general, microbial life in the rhizosphere has been linked to certain genetic factors (e.g. genomes with increased potential for chemotaxis and carbohydrate metabolism) that reflect an adaptation to a plant-associated lifestyle [11]. Selection for certain microbial traits in the rhizosphere is heavily influenced by the evolutionary history of the symbiosis and plant host, which secretes a diverse set of carbon-rich root exudates (e.g. sugars, amino acids, organic acids, phenolics, fatty acids) to recruit microbes to its roots [12–16].

To date, most biochemical studies on root microbiome recruitment have focused on individual exudate compounds, such as specific small signaling molecules, antibiotics, and plant hormones, enabling us to parse the roles of these compounds in specific plant–microbe interactions (e.g. rhizobia and mycorrhizae) [17–19]. However, these metabolites represent just a fraction of the exudates identified in the rhizosphere [20]. Plant glycan profiling has helped identify several host-derived substrates, signals, and toxins that either help or hinder associations with soil microbes [21–25]. While there remain many classes of exudates yet to be linked to the recruitment of specific microbes, experiments testing the ability of microbes to take up exudate compounds individually (e.g. using Biolog plates) or from a synthetic exudate cocktail (e.g. via exometabolomics) are revealing potential cross-feeding interactions [20,26]. Recent technological advances in metabolomics may now enable more comprehensive profiling of root exudates, which vary with host genotype, plant age, phytohormone signaling, and environmental factors — all of which may feedback on one another to influence plant responses to stress [27]. Such comprehensive profiling introduces a new challenge, as chemical identification of unknowns implicated in plant–microbe interactions is highly dependent on the availability of standards and quality of the databases supporting the analyses [28]. Even still, unknown compounds can provide valuable insight in a comparative context. Additionally, one could imagine that compounds that help plants deal with osmotic stress and recruitment/retainment of microbes under stress, like betaines [20] and benzoxazinoids [29], may be important under climate change. As we build our databases, research linking the chemical interactions observed in single-strain experiments with those found in whole microbial communities should be prioritized [15].

## Microbe-mediated mechanisms

It is now well documented that microbial community diversity and composition are important aspects of plant health [10,30–32]. An increase in microbial diversity can expand the range of functions available to plant hosts, potentially improving pathogen protection, nutrient acquisition, phytohormone production, and overall stress tolerance, as well as altering the microbe–microbe interactions that can occur [33–35]. These interactions can be dynamic with some microbes competing for space and resources, while others co-operate or engage in metabolite exchange. However, predicting these community functions from the traits of single taxa and their pairwise interactions is not straightforward, and may have implications for which approach, bottom-up or top-down, is best for microbial consortia selection [36].

Untangling this complexity requires us to consider the presence and consequence of higher-order interactions. Higher-order interactions occur when the function of a pair of species is altered by or dependent on the presence of a third or more species and are especially important to consider given that metabolic activities are often divided among microbial guilds [37,38]. Under abiotic stress, altered soil conditions can modify the ecological interactions that occur across time and space which in turn can modulate species abundance or the expression of functionally relevant genes [32,36]. For example, in dense communities like soil, abiotic stress may increase resource competition which could, in turn, drive changes in biochemical transformations and/or metabolic efficiency in biofilm-forming taxa [39]. These complex, higher-order interactions may be important for maintaining balance among microbial community members that contribute to plant health under stress.

Despite increasing evidence of the importance of microbe–microbe interactions for plant health, we still know little about the mechanisms behind these interactions. Deciphering the molecular communication mechanisms regulating a microbe's influence will be key to harnessing the power of beneficial microbes. Thus, research into microbially produced metabolites associated with toxicity, defense, antibiotic resistance, and quorum-sensing is needed [33]. Considering the time frame over which these interactions occur may also help reveal the molecular mechanisms driving functional shifts in metabolomic profiles. Over short time scales changes in transcriptional regulation may be most important, whereas over longer time scales changes in abundance, as well as genetic changes associated with mutation and horizontal gene transfer, may dominate [39]. A well-known example is the loss-of-function mutation driven by iron limitation associated with siderophore biosynthesis and iron-scavenging in the plant pathogen, *Pseudomonas aeruginosa.* Competition among cheater (cannot produce siderophores but can scavenge them) and non-cheater populations reduced the fitness advantage of the pathogen, promoting the coexistence of many unrelated microbial taxa [40]. Secondary metabolites associated with microbial competition (and subsequent plant pathogen resistance), like siderophores, are often part of biosynthetic gene clusters — many of which remain cryptic because their expression relies on community interactions. Uncovering these clusters by combining metagenomics and metabolomics and identifying the circumstances required for their expression should be a top priority under climate change [41–43]. For instance, temperature stress was identified as a strong selective pressure on both the competition within and cross-feeding between denitrifying and fermentative microbial communities [44] — similar effects are likely to happen in soil microbiomes under climate change. In fact, a survey of over 800 microbial communities from diverse habitats found that interspecies metabolic exchanges are widespread and their metabolic dependency on one another drives species co-occurrences [45]. In sum, adaptations associated with cross-feeding or competition for resources may be missed if we only consider pairwise interactions or single-species evolution. Finally, manipulating the assembly of plant-associated strains may also help reveal historically contingent microbial functions [46]. Given that the early stages of microbial recruitment are likely influenced by stochastic processes, we cannot rule out the possibility of certain taxa driving priority effects during community assembly and thereby affecting plant health later on [47]. Rebuilding a microbiome after disturbances such as wildfires, extreme drought, and extreme flooding (all of which are expected to increase under climate change) might be particularly sensitive to historical contingencies.

## Environment-mediated mechanisms

Researchers have faced limits to independently varying the multiple abiotic stressors associated with climate change both in the field and laboratory. They have also been challenged by measuring climate change effects at both microbe and plant-relevant temporal and spatial scales [9]. Plants and their microbiomes may vary in their timing and ability to respond to certain stressors, based on differences in their physiology, sensitivity to abiotic factors, and growth rates [48]. These differences could create a disconnect between plant development and microbial recruitment, alter nutrient allocation and exchange, and ultimately change microbial composition, function, or the direction (positive or negative) and strength of interactions [49,50].

 It is also likely that plant adaptations to stressors arise due to a complex combination of plant–microbe–environment factors [7,51]. Under this scenario, a change in environmental conditions can directly alter the traits of both plants (e.g. water use efficiency and photosynthesis) and soil microbes (e.g. biofilm formation and osmoprotection) which enable them to respond to stress and prevent death [7]. In addition, changes in these traits can indirectly influence the traits responsible for the formation of a beneficial plant–microbe symbiosis (e.g. root exudation, phytohormone and antioxidant production, nutrient acquisition). For example, the microbial enzyme 1-aminocyclopropane-1-carboxylate (ACC) deaminase responsible for stress protection is only induced once a plant host has experienced sufficient environmental stress to accumulate the ethylene precursor, ACC, in its tissues [52]. As a result, ACC deaminase protects plants from ethylene accumulation which would otherwise lead to senescence [53] and can also indirectly drive a beneficial change in root phenotype [54].

The dynamic nature of plant–microbe–environment interactions is especially important for larger ecosystem processes likely affected by climate change, such as carbon cycling. Some studies predict that elevated levels of carbon dioxide will enhance plant biomass and the movement of carbon to the roots, in turn stimulating the microbial decomposition of soil organic matter which could ultimately decrease carbon stocks [55,56]. However, this process is highly dependent on the plant species involved [57] and the carbon use efficiency of the microbiome — itself a collective of diverse metabolisms each reliant on environmental conditions and context-dependent exchanges between species [58,59]. Thus, changes in microbial community composition may not only influence the current efficiency of carbon cycling, but also the stability and resilience of these processes to continued climate change disturbances. To best predict these dynamic plant–microbiome processes and improve our climate models, it will be crucial to understand how abiotic stressors associated with climate change trigger changes in biochemical reactions leading to mismatches in microbial responses and interactions.

## Tools for untangling plant–microbe interactions

Pinpointing which stress responses are environment, plant, or microbe-mediated will require varied approaches. Top-down approaches involving community reduction and bottom-up approaches involving community assembly from isolates will be critical for validating these predictions [60]. Given this, we believe that researchers will increasingly rely on tools to structure and standardize plant–microbiome experiments such as fabricated ecosystems and synthetic microbial communities (SynComs) (Figure 1B).

One of the biggest challenges in identifying environment-mediated mechanisms under climate change is our inability to independently manipulate abiotic factors in the field and even in the laboratory. Precise, reproducible control of multiple abiotic factors is critical for detailed analyses of plant and microbe-mediated mechanisms of environmental response. EcoFABs are an example of a type of ‘*fab*ricated *eco*system’ designed to create controllable, standardized laboratory environments for the mechanistic study of plant–microbe interactions [61]. As compact, flow-through devices, EcoFABs allow for dynamic, reproducible, and scalable sampling of root microbiome composition, root chemistry (via proteomics and metabolomics), and root morphology/microbial localization (via fluorescent microscopy) [62].

EcoFABs can also help unravel the complexity of microbial community dynamics in the rhizosphere. They combine the benefits of existing tools such as binary plant–microbial association assays (trait assays conducted between a single microbe and plant host [63,64]) and RootChip-type devices (microfluidic devices for live-cell imaging and root phenotyping [65]) into a single device. In such a fully defined system, SynComs can be manipulated to add and subtract components of the ecosystem in real time, for example, to test how increasing microbial diversity might expand the range of plant–microbe and microbe–microbe interactions that can occur [60]. This design also allows researchers to discover and test the presence of higher order interactions in the plant microbiome [36], which could change ecological functional landscapes or result in novel community functions [60].

Most importantly, when paired with omic techniques, EcoFABs become powerful systems for uncovering how beneficial plant microbiomes are formed through plant–microbe–environment interactions [66]. To explore plant-mediated mechanisms such as root exudate composition or microbe-mediated communication signals, EcoFABs can be combined with high-throughput metabolomics. Of course, deciphering this metabolic cross-talk *in situ* and exploring its downstream effects on rhizosphere assembly and plant phenotype will require creative strategies to distinguish plant and microbial metabolites [66]. These include experimental techniques such as isotopic labeling [67] and spatially resolved omics [68,69] (Figure 1C), and a diverse suite of bioinformatic tools linking genomes to metabolites and individual metabolites to whole community metabolic models [70,71] (Table 1). So far, EcoFABs have been combined with some of these tools to test predictions about plant–microbe metabolite exchange under varied phosphate conditions [72].

**Table 1.** Bioinformatic tools for biochemical exploration of plant–microbe interactions from metabolites, genomes, and communities

Bioinformatic tool	Description
*Genome-metabolite identification/annotation*	
MAGI	Analysis that scores the consensus between genomic and metabolomic datasets via biochemical reaction networks to provide better quality metabolite identifications [70]
Metabolome searcher	Uses organism's genome to identify metabolites and metabolic pathways not currently found in compound databases [76]
antiSMASH	Pipeline for secondary metabolite genome mining widely used for identifying biosynthetic gene clusters [77] (repository for clusters with known functions: MIBiG [78])
KEGG, BioCyc	Databases for uncovering biochemical functions and pathways from genome sequences and other molecular datasets [79,80]
*Metabolic modeling*	
KBase	Systems biology platform for complex analyses of genomes and biochemistry including reconstructing and analyzing individual and community metabolic models [81]
ModelSEED, RAVEN, COBRA	High-throughput generation and analysis of genome-scale (single-organism) metabolic models [82–84]
*Integrated pipelines*	
GOLD and IMG/M	Database and comparative analysis tool for amplicons, annotated isolate genomes, metagenomes, and metatranscriptomes [85,86]
GNPS dashboard	Integrates metabolomic and proteomic datasets from various sources for visualization and analysis [87,88]

While EcoFABs and laboratory growth chambers allow researchers to precisely control many abiotic variables, their small size limits the range of plants that can be studied. Laboratory-based mesocosms, such as those employing EcoPOD fabricated ecosystems (inspired by a ‘pod’ as ‘a self-contained unit’), present a solution. They provide researchers with precise environmental control, the ability to colonize with specific microbes or SynComs, as well as the size and flexibility to work with a variety of plants that previously could only be studied in the field [66,73]. Researchers can take intact soil monoliths from the field and study the effects of environmental changes on both plant and soil organisms as a functional ecosystem, as has been tested in the outdoor mesocosm, PEATCosm [66,74]. In this way, EcoPODs potentially provide more precise manipulation of environmental conditions and *in situ* measurements of ecosystem dynamics (e.g. biogeochemical fluxes via sensor technology) and plant growth (e.g. biomass over time via hyperspectral cameras) than field experiments. In other mesocosms, transparent soil systems have been combined with molecular probes and fluorescently labeled bacteria to map microbial colonization and glycan distribution across plant development [75]. These experiments could be adapted for use in EcoPODs, which would enable researchers to not only track specific molecules involved in plant–microbe interactions, but also precisely measure how the transport and assimilation of these molecules correlates with environmental conditions. Conducting plant transcriptomics, shotgun metagenomics and metatranscriptomics on rhizosphere soil within EcoPOD experiments could also expand our understanding of the short- and long-term effects of abiotic stressors on plant–microbe symbioses that do not involve physical adherence to plant roots.

## Conclusion

Facilitating plant adaptation to climate change will require a systems-based approach that accounts for the multi-dimensional nature of plant–microbe–environment interactions. Omics techniques, fabricated ecosystems, and SynComs afford researchers the opportunity to embrace this complexity and use it to create strategies for stress tolerance. These strategies could include manipulations such as providing ‘climate-adapted’ soil microbes to plants experiencing new stress or ‘plant-adapted’ microbes to plants growing in new regions made accessible by climate changes. While teasing apart the relative contributions of the host, its microbes, and the environment in plant adaption to climate change will not be easy nor likely yield a one-size-fits-all explanation, ignoring the complexity limits our ability to create meaningful, sustainable solutions.

## Abbreviations

ACC: aminocyclopropane-1-carboxylate
SynComs: synthetic microbial communities

## References

[1] Nolan, C., Overpeck, J.T., Allen, J.R.M., Anderson, P.M., Betancourt, J.L., Binney, H.A., et al. (2018) Past and future global transformation of terrestrial ecosystems under climate change. Science 361, 920–923 10.1126/science.aan5360 30166491

[2] Pugnaire, F.I., Morillo, J.A., Peñuelas, J., Reich, P.B., Bardgett, R.D., Gaxiola, A. et al. (2019) Climate change effects on plant-soil feedbacks and consequences for biodiversity and functioning of terrestrial ecosystems. Sci. Adv. 5, eaaz1834 10.1126/sciadv.aaz1834 31807715 PMC6881159

[3] Song, Y. and Haney, C.H. (2021) Drought dampens microbiome development. Nat. Plants 7, 994–995 10.1038/s41477-021-00977-z 34294908

[4] Santos-Medellín, C., Liechty, Z., Edwards, J., Nguyen, B., Huang, B., Weimer, B.C. et al. (2021) Prolonged drought imparts lasting compositional changes to the rice root microbiome. Nat. Plants 7, 1065–1077 10.1038/s41477-021-00967-1 34294907

[5] Compant, S., Van Der Heijden, M.G.A. and Sessitsch, A. (2010) Climate change effects on beneficial plant–microorganism interactions. FEMS Microbiol. Ecol. 73, 197–214 10.1111/j.1574-6941.2010.00900.x 20528987

[6] Haichar, F.E.Z., Cernava, T., Liu, J. and Timm, C.M. (2021) Editorial: novel insights into the response of the plant microbiome to abiotic factors. Front. Plant Sci. 12, 996 10.3389/fpls.2021.607874 PMC8193938 34122462

[7] de Vries, F. T., Griffiths, R. I., Knight, C. G., Nicolitch, O. and Williams, A. (2020) Harnessing rhizosphere microbiomes for drought-resilient crop production. Science 368, 270–274 10.1126/science.aaz5192 32299947

[8] Cavicchioli, R., Ripple, W.J., Timmis, K.N., Azam, F., Bakken, L.R., Baylis, M., et al. (2019) Scientists’ warning to humanity: microorganisms and climate change. Nat. Rev. Microbiol. 17, 569–586 10.1038/s41579-019-0222-5 31213707 PMC7136171

[9] Rudgers, J.A., Afkhami, M.E., Bell-Dereske, L., Chung, Y.A., Crawford, K.M., Kivlin, S.N. et al. (2020) Climate disruption of plant–microbe interactions. Annu. Rev. Ecol. Evol. 51, 561–586 10.1146/annurev-ecolsys-011720-090819

[10] Hartman, K. and Tringe, S.G. (2019) Interactions between plants and soil shaping the root microbiome under abiotic stress. Biochem. J. 476, 2705–2724 10.1042/BCJ20180615 31654057 PMC6792034

[11] Levy, A., Salas Gonzalez, I., Mittelviefhaus, M., Clingenpeel, S., Herrera Paredes, S., Miao, J., et al. (2018) Genomic features of bacterial adaptation to plants. Nat. Genet. 50, 138–150 10.1038/s41588-017-0012-9 PMC5957079 29255260

[12] Williams, A. and de Vries, F.T. (2020) Plant root exudation under drought: implications for ecosystem functioning. New Phytol. 225, 1899–1905 10.1111/nph.16223 31571220

[13] Vives-Peris, V., de Ollas, C., Gómez-Cadenas, A. and Pérez-Clemente, R.M. (2020) Root exudates: from plant to rhizosphere and beyond. Plant Cell Rep. 39, 3–17 10.1007/s00299-019-02447-5 31346716

[14] Sasse, J., Martinoia, E. and Northen, T. (2018) Feed your friends: do plant exudates shape the root microbiome? Trends Plant Sci. 23, 25–41 10.1016/j.tplants.2017.09.003 29050989

[15] O'Banion, B., O'Neal, L., Alexandre, G. and Lebeis, S. (2019) Bridging the gap between single-strain and community-level plant–microbe chemical interactions. Mol. Plant Microbe Interact. 33, 124–134 10.1094/MPMI-04-19-0115-CR 31687914

[16] Hoeksema, J.D., Bever, J.D., Chakraborty, S., Chaudhary, V.B., Gardes, M., Gehring, C.A., et al. (2018) Evolutionary history of plant hosts and fungal symbionts predicts the strength of mycorrhizal mutualism. Commun. Biol. 1, 116 10.1038/s42003-018-0120-9 30271996 PMC6123707

[17] Li, B., Li, Y.-Y., Wu, H.-M., Zhang, F.-F., Li, C.-J., Li, X.-X. et al. (2016) Root exudates drive interspecific facilitation by enhancing nodulation and N2 fixation. Proc. Natl Acad. Sci. U.S.A. 113, 6496–6501 10.1073/pnas.1523580113 27217575 PMC4988560

[18] Bonfante, P. and Genre, A. (2010) Mechanisms underlying beneficial plant–fungus interactions in mycorrhizal symbiosis. Nat. Commun. 1, 48 10.1038/ncomms1046 20975705

[19] Steinkellner, S., Lendzemo, V., Langer, I., Schweiger, P., Khaosaad, T., Toussaint, J.-P. et al. (2007) Flavonoids and strigolactones in root exudates as signals in symbiotic and pathogenic plant–fungus interactions. Molecules 12, 1290–1306 10.3390/12071290 17909485 PMC6149470

[20] Zhalnina, K., Louie, K.B., Hao, Z., Mansoori, N., da Rocha, U.N., Shi, S., et al. (2018) Dynamic root exudate chemistry and microbial substrate preferences drive patterns in rhizosphere microbial community assembly. Nat. Microbiol. 3, 470–480 10.1038/s41564-018-0129-3 29556109

[21] Xu, L., Naylor, D., Dong, Z., Simmons, T., Pierroz, G., Hixson, K.K., et al. (2018) Drought delays development of the sorghum root microbiome and enriches for monoderm bacteria. Proc. Natl Acad. Sci. U.S.A. 115, E4284–E4293 10.1073/pnas.1717308115 29666229 PMC5939072

[22] Wanke, A., Malisic, M., Wawra, S. and Zuccaro, A. (2021) Unraveling the sugar code: the role of microbial extracellular glycans in plant–microbe interactions. J. Exp. Bot. 72, 15–35 10.1093/jxb/eraa414 32929496 PMC7816849

[23] Grondin, J.M., Tamura, K., Déjean, G., Abbott, D.W. and Brumer, H. (2017) Polysaccharide utilization loci: fueling microbial communities. J. Bacteriol. 19, e00860-16 10.1128/JB.00860-16 PMC5512228 28138099

[24] Koroney, A.S., Plasson, C., Pawlak, B., Sidikou, R., Driouich, A., Menu-Bouaouiche, L. et al. (2016) Root exudate of *Solanum tuberosum* is enriched in galactose-containing molecules and impacts the growth of *Pectobacterium atrosepticum*. Ann. Bot. 118, 797–808 10.1093/aob/mcw128 27390353 PMC5055634

[25] Castilleux, R., Plancot, B., Ropitaux, M., Carreras, A., Leprince, J., Boulogne, I. et al. (2018) Cell wall extensins in root–microbe interactions and root secretions. J. Exp. Bot. 69, 4235–4247 10.1093/jxb/ery238 29945246

[26] Swenson, T.L., Karaoz, U., Swenson, J.M., Bowen, B.P. and Northen, T.R. (2018) Linking soil biology and chemistry in biological soil crust using isolate exometabolomics. Nat. Commun. 9, 19 10.1038/s41467-017-02356-9 29296020 PMC5750228

[27] Haldar, S. and Sengupta, S. (2015) Plant-microbe cross-talk in the rhizosphere: insight and biotechnological potential. Open Microbiol. J. 9, 1–7 10.2174/1874285801509010001 25926899 PMC4406998

[28] Bowen, B.P. and Northen, T.R. (2010) Dealing with the unknown: metabolomics and metabolite atlases. J. Am. Soc. Mass Spectrom. 21, 1471–1476 10.1016/j.jasms.2010.04.003 20452782

[29] Hu, L., Robert, C.A.M., Cadot, S., Zhang, X., Ye, M., Li, B., et al. (2018) Root exudate metabolites drive plant-soil feedbacks on growth and defense by shaping the rhizosphere microbiota. Nat. Commun. 9, 2738 10.1038/s41467-018-05122-7 30013066 PMC6048113

[30] Berg, G., Rybakova, D., Grube, M. and Köberl, M. (2016) The plant microbiome explored: implications for experimental botany. J. Exp. Bot. 67, 995–1002 10.1093/jxb/erv466 26547794 PMC5395086

[31] Cordovez, V., Dini-Andreote, F., Carrión, V.J. and Raaijmakers, J.M. (2019) Ecology and evolution of plant microbiomes. Annu. Rev. Microbiol. 73, 69–88 10.1146/annurev-micro-090817-062524 31091418

[32] Trivedi, P., Leach, J.E., Tringe, S.G., Sa, T. and Singh, B.K. (2020) Plant–microbiome interactions: from community assembly to plant health. Nat. Rev. Microbiol. 18, 607–621 10.1038/s41579-020-0412-1 32788714

[33] Berlanga-Clavero, M.V., Molina-Santiago, C., de Vicente, A. and Romero, D. (2020) More than words: the chemistry behind the interactions in the plant holobiont. Environ. Microbiol. 22, 4532–4544 10.1111/1462-2920.15197 32794337

[34] Hassani, M.A., Durán, P. and Hacquard, S. (2018) Microbial interactions within the plant holobiont. Microbiome 6, 58 10.1186/s40168-018-0445-0 29587885 PMC5870681

[35] Khare, E., Tyagi, S. and Patil, K.S. (2020) Chapter 5 - language of plant-microbe-microbe interactions in rhizospheric ecosystems. In Molecular Aspects of Plant Beneficial Microbes in Agriculture (Sharma, V., Salwan, R. and Al-Ani, L.K.T., eds.), pp. 59–76, Academic Press, Cambridge, MA, USA

[36] Sanchez-Gorostiaga, A., Bajić, D., Osborne, M.L., Poyatos, J.F. and Sanchez, A. (2019) High-order interactions distort the functional landscape of microbial consortia. PLOS Biol. 17, e3000550 10.1371/journal.pbio.3000550 31830028 PMC6932822

[37] Hamonts, K., Trivedi, P., Garg, A., Janitz, C., Grinyer, J., Holford, P. et al. (2018) Field study reveals core plant microbiota and relative importance of their drivers. Environ. Microbiol. 20, 124–140 10.1111/1462-2920.14031 29266641

[38] Durán, P., Thiergart, T., Garrido-Oter, R., Agler, M., Kemen, E., Schulze-Lefert, P. et al. (2018) Microbial interkingdom interactions in roots promote arabidopsis survival. Cell 175, 973–983 10.1016/j.cell.2018.10.020 30388454 PMC6218654

[39] Cao, X., Hamilton, J.J. and Venturelli, O.S. (2019) Understanding and engineering distributed biochemical pathways in microbial communities. Biochemistry 58, 94–107 10.1021/acs.biochem.8b01006 30457843 PMC6733022

[40] Leinweber, A., Fredrik Inglis, R. and Kümmerli, R. (2017) Cheating fosters species co-existence in well-mixed bacterial communities. ISME J. 11, 1179–1188 10.1038/ismej.2016.195 28060362 PMC5437929

[41] Tracanna, V., Ossowicki, A., Petrus, M. L. C., Overduin, S., Terlouw, B. R., Lund, G., et al. (2021) Dissecting disease-suppressive rhizosphere microbiomes by functional amplicon sequencing and 10× metagenomics. mSystems 6, e0116-20. 10.1128/mSystems.01116-20 PMC8269251 34100635

[42] Zhou, L., Song, C., Li, Z. and Kuipers, O.P. (2021) Antimicrobial activity screening of rhizosphere soil bacteria from tomato and genome-based analysis of their antimicrobial biosynthetic potential. BMC Genom. 22, 29 10.1186/s12864-020-07346-8 PMC7789753 33413100

[43] Mendes, R., Kruijt, M., de Bruijn, I., Dekkers, E., van der Voort, M., Schneider, J.H.M., et al. (2011) Deciphering the rhizosphere microbiome for disease-suppressive bacteria. Science 332, 1097–1100 10.1126/science.1203980 21551032

[44] Hanke, A., Berg, J., Hargesheimer, T., Tegetmeyer, H.E., Sharp, C.E. and Strous, M. (2016) Selective pressure of temperature on competition and cross-feeding within denitrifying and fermentative microbial communities. Front. Microbiol. 6, 1461 10.3389/fmicb.2015.01461 26779132 PMC4703780

[45] Zelezniak, A., Andrejev, S., Ponomarova, O., Mende, D.R., Bork, P. and Patil, K.R. (2015) Metabolic dependencies drive species co-occurrence in diverse microbial communities. Proc. Natl Acad. Sci. U.S.A. 112, 6449–6454 10.1073/pnas.1421834112 25941371 PMC4443341

[46] Leopold, D.R. and Busby, P.E. (2020) Host genotype and colonist arrival order jointly govern plant microbiome composition and function. Curr. Biol. 30, 3260–3266.e5 10.1016/j.cub.2020.06.011 32679100

[47] Wei, Z., Gu, Y., Friman, V.-P., Kowalchuk, G.A., Xu, Y., Shen, Q. et al. (2019) Initial soil microbiome composition and functioning predetermine future plant health. Sci. Adv. 5, eaaw0759 10.1126/sciadv.aaw0759 31579818 PMC6760924

[48] Van der Putten, W.H. (2012) Climate change, aboveground-belowground interactions, and species’ range shifts. Annu. Rev. Ecol. Evol. 43, 365–383 10.1146/annurev-ecolsys-110411-160423

[49] Nazir, R., Warmink, J.A., Boersma, H. and Van Elsas, J.D. (2009) Mechanisms that promote bacterial fitness in fungal-affected soil microhabitats. FEMS Microbiol. Ecol. 71, 169–185 10.1111/j.1574-6941.2009.00807.x 20002182

[50] Leifheit, E.F., Verbruggen, E. and Rillig, M.C. (2015) Arbuscular mycorrhizal fungi reduce decomposition of woody plant litter while increasing soil aggregation. Soil Biol. Biochem. 81, 323–328 10.1016/j.soilbio.2014.12.003

[51] Hou, S., Wolinska, K.W. and Hacquard, S. (2021) Microbiota-root-shoot-environment axis and stress tolerance in plants. Curr. Opin. Plant Biol. 62, 102028 10.1016/j.pbi.2021.102028 33713892

[52] Singh, R., Shelke, G., Kumar, A. and Jha, P. (2015) Biochemistry and genetics of ACC deaminase: a weapon to “stress ethylene” produced in plants. Front. Microbiol. 6, 937 10.3389/fmicb.2015.00937 26441873 PMC4563596

[53] Glick, B.R., Cheng, Z., Czarny, J. and Duan, J. (2007) Promotion of plant growth by ACC deaminase-producing soil bacteria. In New Perspectives and Approaches in Plant Growth-Promoting Rhizobacteria Research (Bakker, P.A.H.M., Raaijmakers, J.M., Bloemberg, G., Höfte, M., Lemanceau, P. and Cooke, B.M., eds.), pp. 329–339, Springer Netherlands, Dordrecht

[54] Glick, B.R. (2005) Modulation of plant ethylene levels by the bacterial enzyme ACC deaminase. FEMS Microbiol. Lett. 251, 1–7 10.1016/j.femsle.2005.07.030 16099604

[55] Adair, C., Reich, E., Trost, P.B., and Hobbie, J.J. and E, S. (2011) Elevated CO2 stimulates grassland soil respiration by increasing carbon inputs rather than by enhancing soil moisture. Glob. Change Biol. 17, 3546–3563 10.1111/j.1365-2486.2011.02484.x

[56] Bengtson, P., Barker, J. and Grayston, S.J. (2012) Evidence of a strong coupling between root exudation, C and N availability, and stimulated SOM decomposition caused by rhizosphere priming effects. Ecol. Evol. 2, 1843–1852 10.1002/ece3.311 22957187 PMC3433989

[57] Jansson, C., Vogel, J., Hazen, S., Brutnell, T. and Mockler, T. (2018) Climate-smart crops with enhanced photosynthesis. J. Exp. Bot. 69, 3801–3809 10.1093/jxb/ery213 30032188

[58] Jansson, J.K. and Hofmockel, K.S. (2020) Soil microbiomes and climate change. Nat. Rev. Microbiol. 18, 35–46 10.1038/s41579-019-0265-7 31586158

[59] Malik, A.A., Martiny, J.B.H., Brodie, E.L., Martiny, A.C., Treseder, K.K. and Allison, S.D. (2020) Defining trait-based microbial strategies with consequences for soil carbon cycling under climate change. ISME J. 14, 1–9 10.1038/s41396-019-0510-0 31554911 PMC6908601

[60] Vorholt, J.A., Vogel, C., Carlström, C.I. and Müller, D.B. (2017) Establishing causality: opportunities of synthetic communities for plant microbiome research. Cell Host Microbe 22, 142–155 10.1016/j.chom.2017.07.004 28799900

[61] Gao, J., Sasse, J., Lewald, K.M., Zhalnina, K., Cornmesser, L.T., Duncombe, T.A. et al. (2018) Ecosystem fabrication (EcoFAB) protocols for the construction of laboratory ecosystems designed to study plant–microbe interactions. J. Vis. Exp. 134, 57170 10.3791/57170 PMC5933423 29708529

[62] Zengler, K., Hofmockel, K., Baliga, N.S., Behie, S.W., Bernstein, H.C., Brown, J.B., et al. (2019) EcoFABs: advancing microbiome science through standardized fabricated ecosystems. Nat. Methods 16, 567–571 10.1038/s41592-019-0465-0 31227812 PMC6733021

[63] Paredes, S.H., Gao, T., Law, T.F., Finkel, O.M., Mucyn, T., Teixeira, P.J.P.L., et al. (2018) Design of synthetic bacterial communities for predictable plant phenotypes. PLOS Biol. 16, e2003962 10.1371/journal.pbio.2003962 29462153 PMC5819758

[64] Bulgarelli, D., Garrido-Oter, R., Münch, P.C., Weiman, A., Dröge, J., Pan, Y. et al. (2015) Structure and function of the bacterial root microbiota in wild and domesticated barley. Cell Host Microbe 17, 392–403 10.1016/j.chom.2015.01.011 25732064 PMC4362959

[65] Grossmann, G., Guo, W.-J., Ehrhardt, D.W., Frommer, W.B., Sit, R.V., Quake, S.R. et al. (2011) The rootChip: an integrated microfluidic chip for plant science. The Plant Cell 23, 4234–4240 10.1105/tpc.111.092577 22186371 PMC3269862

[66] Singer, E., Vogel, J.P., Northen, T., Mungall, C.J. and Juenger, T.E. (2021) Novel and emerging capabilities that can provide a holistic understanding of the plant root microbiome. Phytobiomes J. 5, 122–132 10.1094/PBIOMES-05-20-0042-RVW

[67] Dunford, E.A. and Neufeld, J.D. (2010) DNA stable-isotope probing (DNA-SIP). J. Vis. Exp. 42, 2027 10.3791/2027 PMC3156007 20729803

[68] Boughton, B. A. and Thinagaran, D. (2018) Mass spectrometry imaging (MSI) for plant metabolomics. In Plant Metabolomics: Methods and Protocols (António, C., ed.), pp. 241–252, Springer, New York, NY 10.1007/978-1-4939-7819-9_17 29761443

[69] Masuda, K., Abouleila, Y., Ali, A., Yanagida, T. and Masujima, T. (2018) Live single-cell mass spectrometry (LSC-MS) for plant metabolomics. In Plant Metabolomics: Methods and Protocols (António, C., ed.), pp. 269–282, Springer, New York, NY 10.1007/978-1-4939-7819-9_19 29761445

[70] Erbilgin, O., Rübel, O., Louie, K.B., Trinh, M., de Raad, M., Wildish, T., et al. (2019) MAGI: a method for metabolite annotation and gene integration. ACS Chem. Biol. 14, 704–714 10.1021/acschembio.8b01107 30896917

[71] Henry, C.S., Bernstein, H.C., Weisenhorn, P., Taylor, R.C., Lee, J.-Y., Zucker, J. et al. (2016) Microbial community metabolic modeling: a community data-Driven network reconstruction. J. Cell. Physiol. 231, 2339–2345 10.1002/jcp.25428 27186840 PMC5132105

[72] Sasse, J., Kant, J., Cole, B.J., Klein, A.P., Arsova, B., Schlaepfer, P., et al. (2019) Multilab EcoFAB study shows highly reproducible physiology and depletion of soil metabolites by a model grass. New Phytol. 222, 1149–1160 10.1111/nph.15662 30585637 PMC6519027

[73] Eisenhauer, N. and Türke, M. (2018) From climate chambers to biodiversity chambers. Front. Ecol. Environ. 16, 136–137 10.1002/fee.1784

[74] Haynes, K.M., Kane, E.S., Potvin, L., Lilleskov, E.A., Kolka, R.K. and Mitchell, C.P.J. (2019) Impacts of experimental alteration of water table regime and vascular plant community composition on peat mercury profiles and methylmercury production. Sci. Total Environ. 682, 611–622 10.1016/j.scitotenv.2019.05.072 31129544

[75] Jones, C.Y., Engelhardt, I., Patko, D., Dupuy, L., Holden, N. and Willats, W.G.T. (2021) High-resolution 3D mapping of rhizosphere glycan patterning using molecular probes in a transparent soil system. Cell Surface 7, 100059 10.1016/j.tcsw.2021.100059 PMC8445887 34557617

[76] Dhanasekaran, A.R., Pearson, J.L., Ganesan, B. and Weimer, B.C. (2015) Metabolome searcher: a high throughput tool for metabolite identification and metabolic pathway mapping directly from mass spectrometry and using genome restriction. BMC Bioinform. 16, 62 10.1186/s12859-015-0462-y PMC4347650 25887958

[77] Blin, K., Shaw, S., Steinke, K., Villebro, R., Ziemert, N., Lee, S.Y. et al. (2019) antiSMASH 5.0: updates to the secondary metabolite genome mining pipeline. Nucleic Acids Res. 47, W81–W87 10.1093/nar/gkz310 31032519 PMC6602434

[78] Kautsar, S.A., Blin, K., Shaw, S., Navarro-Muñoz, J.C., Terlouw, B.R., van der Hooft, J.J.J., et al. (2020) MIBig 2.0: a repository for biosynthetic gene clusters of known function. Nucleic Acids Res. 48, D454–D458 10.1093/nar/gkz882 31612915 PMC7145714

[79] Kanehisa, M. (1997) A database for post-genome analysis. Trends Genet. 13, 375–376 10.1016/S0168-9525(97)01223-7 9287494

[80] Karp, P.D., Billington, R., Caspi, R., Fulcher, C.A., Latendresse, M., Kothari, A., et al. (2019) The BioCyc collection of microbial genomes and metabolic pathways. Brief Bioinform. 20, 1085–1093 10.1093/bib/bbx085 29447345 PMC6781571

[81] Arkin, A.P., Cottingham, R.W., Henry, C.S., Harris, N.L., Stevens, R.L., Maslov, S., et al. (2018) KBase: the United States department of energy systems biology knowledgebase. Nat. Biotechnol. 36, 566–569 10.1038/nbt.4163 29979655 PMC6870991

[82] Heirendt, L., Arreckx, S., Pfau, T., Mendoza, S.N., Richelle, A., Heinken, A., et al. (2019) Creation and analysis of biochemical constraint-based models using the COBRA toolbox v.3.0. Nat. Protoc. 14, 639–702 10.1038/s41596-018-0098-2 30787451 PMC6635304

[83] Henry, C.S., DeJongh, M., Best, A.A., Frybarger, P.M., Linsay, B. and Stevens, R.L. (2010) High-throughput generation, optimization and analysis of genome-scale metabolic models. Nat. Biotechnol. 28, 977–982 10.1038/nbt.1672 20802497

[84] Agren, R., Liu, L., Shoaie, S., Vongsangnak, W., Nookaew, I. and Nielsen, J. (2013) The RAVEN toolbox and Its Use for generating a genome-scale metabolic model for *penicillium chrysogenum*. PLOS Comput. Biol. 9, e1002980 10.1371/journal.pcbi.1002980 23555215 PMC3605104

[85] Mukherjee, S., Stamatis, D., Bertsch, J., Ovchinnikova, G., Katta, H.Y., Mojica, A. et al. (2019) Genomes onLine database (GOLD) v.7: updates and new features. Nucleic Acids Res. 47, D649–D659 10.1093/nar/gky977 30357420 PMC6323969

[86] Chen, I.-M.A., Chu, K., Palaniappan, K., Ratner, A., Huang, J., Huntemann, M., et al. (2021) The IMG/M data management and analysis system v.6.0: new tools and advanced capabilities. Nucleic Acids Res. 49, D751–D763 10.1093/nar/gkaa939 33119741 PMC7778900

[87] Wang, M., Carver, J.J., Phelan, V.V., Sanchez, L.M., Garg, N., Peng, Y., et al. (2016) Sharing and community curation of mass spectrometry data with GNPS. Nat. Biotechnol. 34, 828–837 10.1038/nbt.3597 27504778 PMC5321674

[88] Petras, D., Phelan, V.V., Acharya, D., Allen, A.E., Aron, A.T., Bandeira, N., et al. (2021) GNPS dashboard: collaborative exploration of mass spectrometry data in the web browser. Nat. Methods 1–3 10.1038/s41592-021-01339-5 34862502 PMC8831450

